# BRAFV600E mutation positive metastatic melanoma in a young woman treated with anti-BRAF/anti MEK combination: a case report

**DOI:** 10.1186/1479-5876-13-S1-P8

**Published:** 2015-01-15

**Authors:** Sara Giovannoni, Federica Urbano, Daniela Modica, Sofia Verkhovskaia, Giuliana Caprio, Silvia Mezi, Enrico Cortesi

**Affiliations:** 1 Oncologia B - Dpt Sc. Radiologiche, Oncologiche, Anatomo Patologiche, Policlinico Umberto I. “Sapienza” Università di Roma, Italy

## Background

The recent Combi-v [1] and Combi-d [2,3], phase III randomized trials, showed, respectively, an OS benefit with Dabrafenib/Trametinib combination versus Vemurafenib and an improvement in PFS and ORR with the same combination versus Dabrafenib alone, in BRAF V600E/K mutation positive metastatic or unresectable cutaneous melanoma. We report the case of a young patient with metastatic melanoma treated with antiBRAF/antiMEK combination.

## Case report

A 36-years woman referred to our institution in March 2014, after the diagnosis of metastatic melanoma. ECOG Performance Status was 1. No comorbidities, no previous melanoma treatment. In May 2011 the patient reported a traumatic event which resulted in the removal of a mole localized in the right thigh. Since August 2013 she noted a swelling in the right area of the groin. It increased constantly causing pain, deambulation problems and limiting daily activities. On January 2014, due to the worsening of the swelling and pain, which resulted in the patient becoming unable to maintain a sitting position, she underwent an ultrasound exam that displayed pathological lymph nodes. A FNAB showed a metastatic localization of melanoma, S 100+++, Hbm 45++. BRAFV600E mutation was detected with Cobas 4800 BRAF mutation test. A CT/PET displayed conglobated pathological lymph nodes in right groin of around 10 cm; right iliac obturator lymph node of 6 cm and osteolytic lesion at sacroiliac articulation. In April 2014, after adequate screening, the patient began Dabrafenib/Trametinib combination under the Compassionate Use program. Just after one month of treatment she reported clinical benefit in terms of deambulation improvement and pain relief. During the second month of therapy the treatment with Dabrafenib was withdrawn for some days, due to two episodes of hyperpyrexia up to 39°C, treated with Paracetamol. Progressively the antiBRAF drug was reintroduced with no further interruption. The CT/PET performed after three and six month of treatment showed a good response to the therapy with a dimensional decrease and SUV reduction of more than 50% in all target lesions. Currently the patient is continuing the treatment; her ECOG PS is 0. She does not require any antalgic drugs. Our aim is obtain the maximum reduction to allow a surgical approach.

## Conclusion

In our case the Dabrafenib/Trametinib (antiBRAF/antiMEK) combination leads to a fast clinical and radiological disease response with a good toxicity profile and a good management of AEs.

Written informed consent was obtained from the patient for publication of this abstract. A copy of the written consent is available for review by the Editor of this journal

**Figure 1:**
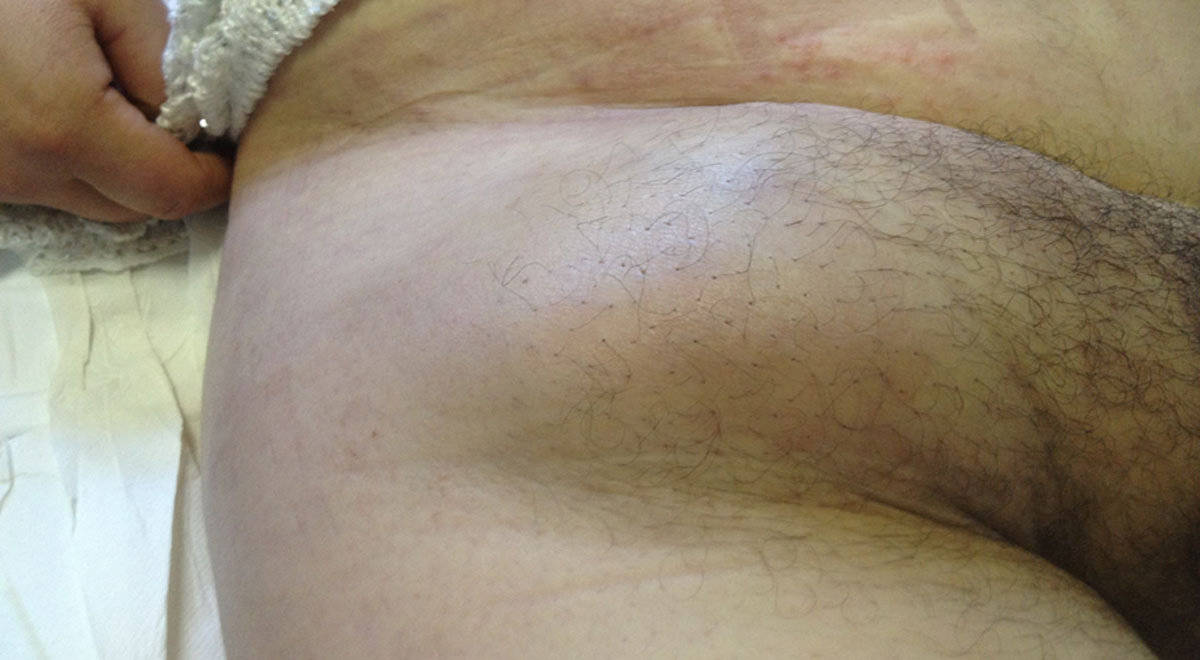
March 2014

**Figure 2:**
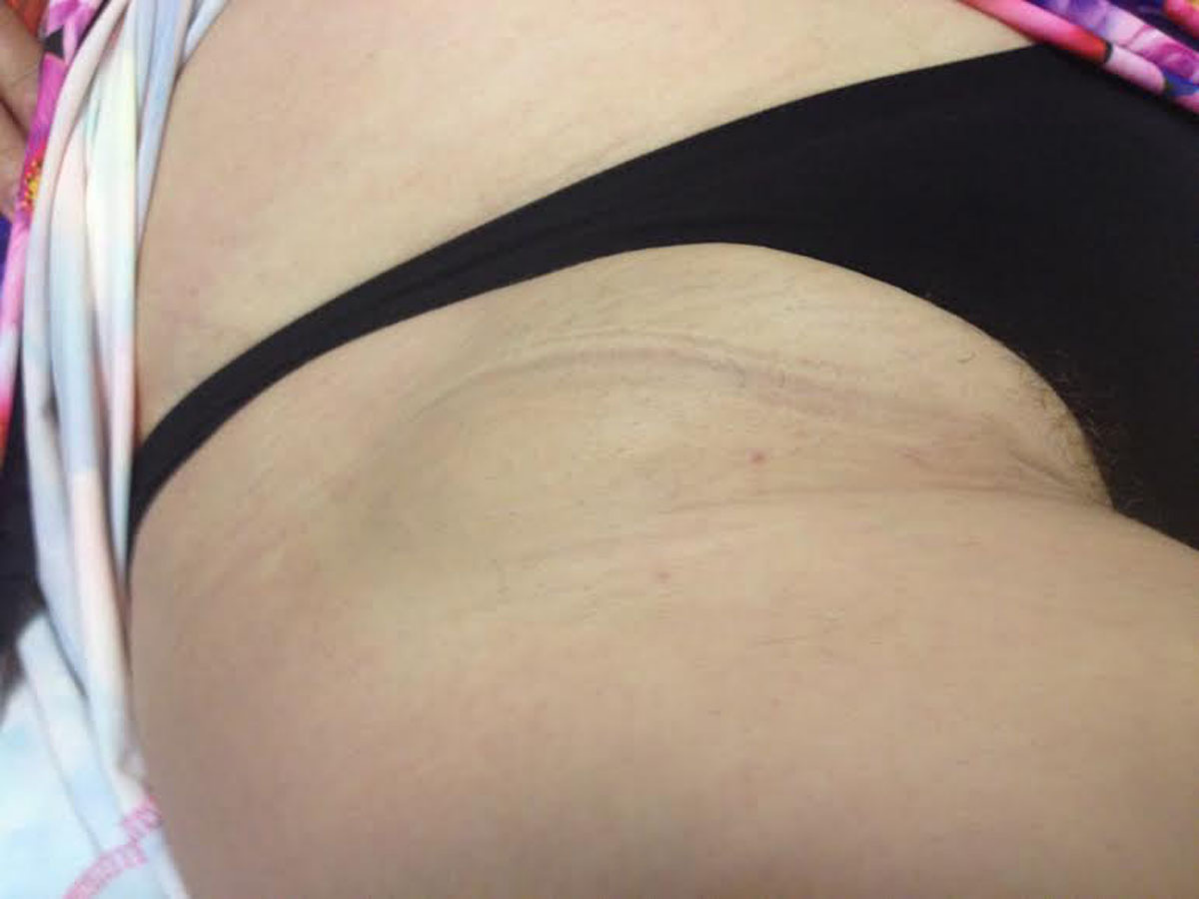
September 2014
